# The changes in ocular torsion after unilateral lateral rectus recession-medial rectus resection for intermittent exotropia

**DOI:** 10.1038/s41598-024-65193-z

**Published:** 2024-06-21

**Authors:** Changyang Liu, Jiasu Liu, Huailin Zhu, Lan Zhang, Mingjun Gao, Siqi Zhang, Qi Zhao

**Affiliations:** Department of Ophthalmology, The Second Hospital of Dalian Medical University, Dalian, 116023 Liaoning China

**Keywords:** Ocular motility disorders, Outcomes research

## Abstract

We aim to explore the alterations of objective ocular torsion after unilateral lateral rectus recession-medial rectus resection (R&R) for intermittent exotropia (IXT). Seventy-two IXT patients undergoing R&R between March and June 2023 were enrolled. Ophthalmological examinations were performed before surgery and at 1 week and 1 month after surgery, mainly including prism and alternate cover test and optical coherence tomography. The mean disc-foveal angle of eyes showing intorsion significantly increased from − 1.5 ± 0.9° preoperatively to 2.0 ± 2.0° at 1 week (*P* = 0.0227) and 2.2 ± 1.6° at 1 month postoperatively (*P* = 0.0054). The mean disc-foveal angle of eyes exhibiting extorsion significantly reduced from 12.8 ± 1.9° preoperatively to 9.8 ± 3.1° at 1 week (*P* < 0.0001) and 9.7 ± 2.7° at 1 month postoperatively (*P* < 0.0001). The improvement of ocular extorsion at postoperative 1 month was more pronounced in patients with extorsion in operative eye compared to those with extorsion in inoperative eye (*P* = 0.0101). The improvement of ocular torsion was observed following R&R for IXT, with a greater effect noted in cases where the surgery was performed on the eye exhibiting extorsion.

## Introduction

The rotation of the eye around its anteroposterior axis, known as ocular torsion, encompasses intorsion and extorsion. The assessment of torsional, horizontal and vertical eye movements provides comprehensive information regarding anomalous ocular movements, which may impact the selection of surgical procedures^1,2^. Objective methods, including optical coherence tomography (OCT) and fundus photography, are available for assessing ocular torsion^3–7^.

Intermittent exotropia (IXT), a common type of divergent strabismus intermittently regulated by fusional mechanisms, appears to be unrelated to ocular torsion. However, fundus photography examinations have revealed the presence of ocular torsion in approximately 30% of children with IXT^8^. Shin et al.^8^ demonstrated a significant positive correlation between the severity of IXT and the degree of ocular torsion, with IXT children exhibiting a larger disc-foveal angle (DFA) than their normal counterparts. Lee et al.^9^ found that ocular torsion could be corrected through either unilateral lateral rectus recession or bilateral lateral rectus recession. Despite unilateral lateral rectus recession-medial rectus resection (R&R) being one of the most popular surgical methods for IXT^10,11^, prior studies have not explored the effects of R&R on ocular torsion. With the increasing use of OCT in clinical settings, numerous studies have assessed ocular torsion using OCT^4,12–14^. In comparison to fundus photography, OCT exhibits superiority in the evaluation of ocular torsion owing to its high levels of efficiency, accuracy, and repeatability^3,4^. This study aims to assess objective ocular torsion in IXT patients using OCT and explore the impact of R&R on ocular torsion.

## Results

In total, 72 patients (144 eyes) were enrolled, of whom 36 were males and 36 females. The mean age at surgery was 9.0 ± 2.5 years (range 5–16 years), and the mean duration of strabismus was 2.67 ± 2.65 years (range 0.02–12 years). The overall DFA was 6.8 ± 4.0° on average, exhibiting a high degree of variability (range − 3.1–17.7°). Ocular torsion was detected in 25 patients (34.7%) through OCT. Among these patients, 22 eyes (15.3%) of 18 individuals (25.0%) were found to have extorsion, while 7 eyes (4.9%) of 7 patients (9.7%) were found to have intorsion. The mean preoperative angle of near deviation was 44.3 ± 13.6 prism diopters (PD) with a range of 20–80 PD, while the mean preoperative angle of distance deviation was 32.8 ± 12.9 PD with a range of 15–66 PD. The mean angle of near deviation was 2.0 ± 4.7 PD and 2.7 ± 4.7 PD at 1 week and 1 month after surgery, respectively, representing a significant reduction compared to preoperative level (all *P* < 0.0001, Supplementary Fig. S1a). Similarly, the mean angle of distance deviation was 0.5 ± 3.4 PD and 1.1 ± 3.5 PD at 1 week and 1 month after surgery, respectively, also demonstrating a significant reduction compared to preoperative level (all *P* < 0.0001, Supplementary Fig. S1b).

There was a significant longitudinal alteration in patient distribution based on qualitative torsion grading (*P* = 0.0067, χ^2^ test for trend), with less patients exhibiting ocular torsion at postoperative 1 month (n = 11, 15.3%).

**Figure 1:**
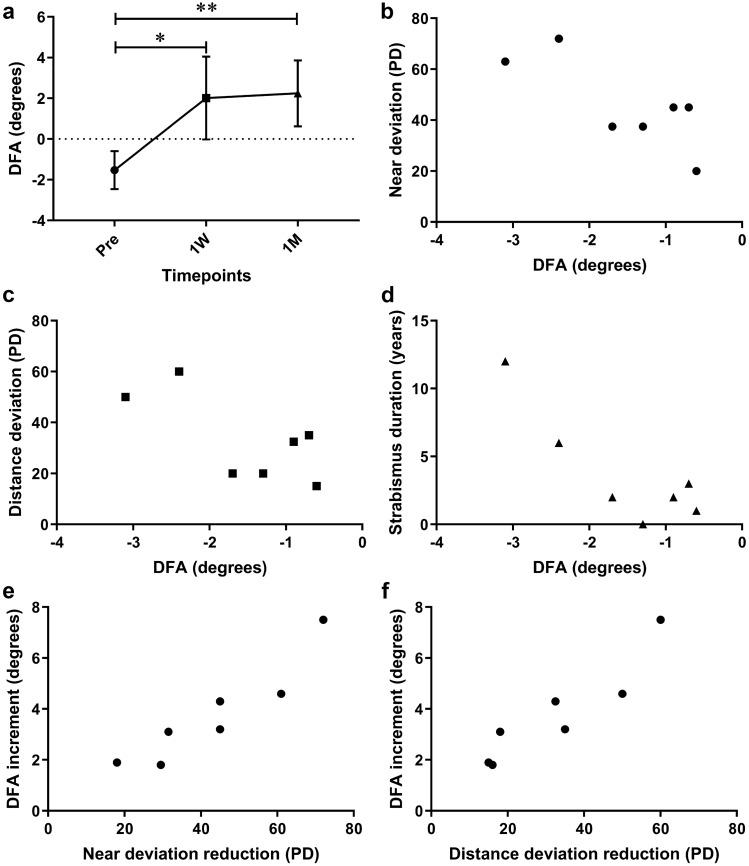
Changes in objective ocular torsion among patients with intorsion. (**a**) Longitudinal changes in disc-foveal angle (DFA) of eyes having intorsion. (**b**) Correlation between DFA and preoperative near deviation. (**c**) Correlation between DFA and preoperative distance deviation. (**d**) Correlation between DFA and strabismus duration. (**e**) Correlation between near deviation reduction and DFA increment at 1 month after surgery. (**f**) Correlation between distance deviation reduction and DFA increment at 1 month after surgery. *P* values for comparisons relative to preoperative level: *P < 0.05 and **P < 0.01; repeated measures (RM) one-way ANOVA with Dunnett’s multiple comparisons test.

Among the patients with ocular intorsion before surgery, the mean preoperative DFA of eyes having intorsion increased significantly from − 1.5 ± 0.9° to 2.0 ± 2.0° at 1 week after surgery (*P* = 0.0173), and further increased to 2.2 ± 1.6° at 1 month (*P* = 0.0040, Fig. 1a). The operative eyes were exactly the ones having intorsion. Pearson correlation analysis indicated significant correlations between the preoperative DFA of eyes having intorsion and the angle of deviation at near, but not at distance, as well as the duration of strabismus (r = − 0.779, *P* = 0.0391, r = − 0.706, *P* = 0.0766, and r = − 0.856, *P* = 0.0139, respectively, Fig. 1b–d). Pearson correlation analysis suggested the correction of near and distance deviation was positively correlated to the increment of DFA at postoperative 1 month (r = 0.925, *P* = 0.0028, and r = 0.922, *P* = 0.0032, respectively, Fig. 1e,f), rather than at postoperative 1 week.

**Figure 2:**
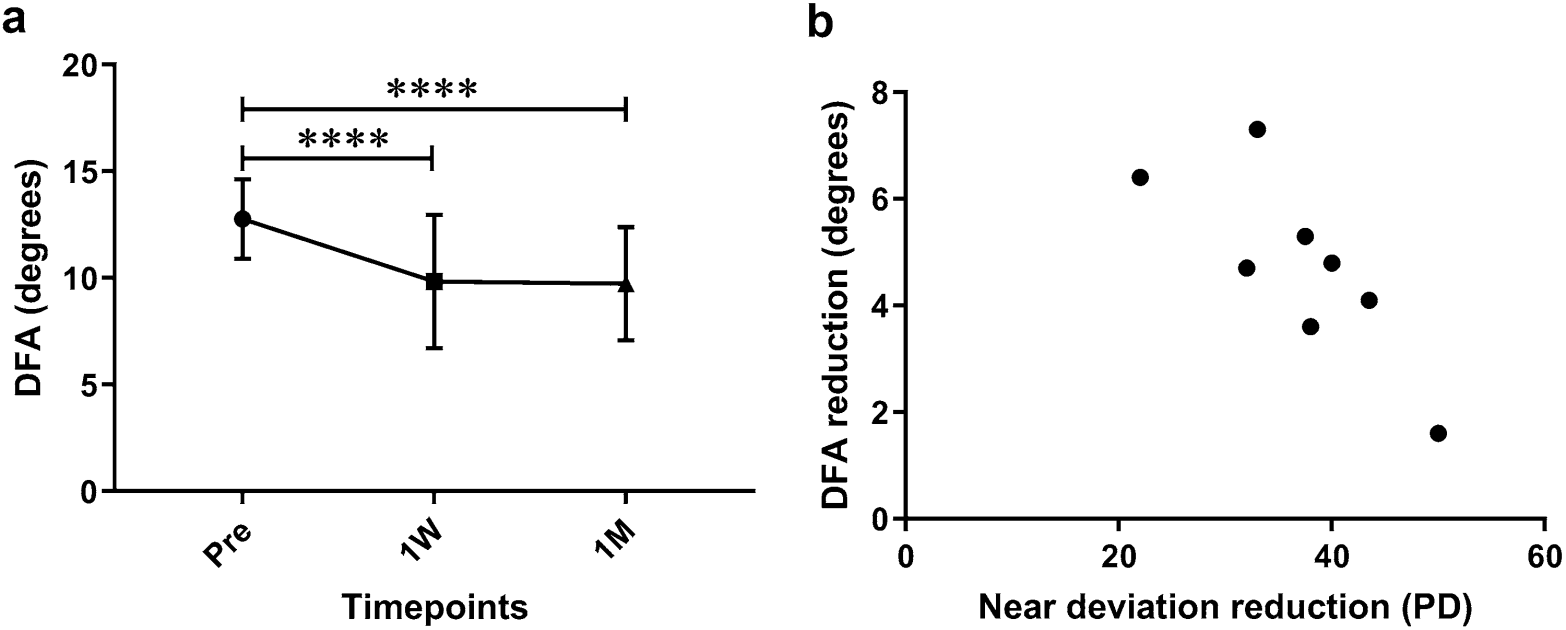
Changes in objective ocular torsion among patients with extorsion. (**a**) Longitudinal changes in DFA of eyes having extorsion. (**b**) Correlation between near deviation reduction and DFA reduction at 1 month after surgery. *P* values for comparisons relative to preoperative level: ****P < 0.0001; RM one-way ANOVA with Dunnett’s multiple comparisons test.

For the 18 patients with ocular extorsion before surgery, the mean DFA of eyes having extorsion decreased from 12.8 ± 1.9° preoperatively to 9.8 ± 3.1° at 1 week postoperatively (*P* < 0.0001) and to 9.7 ± 2.7° at 1 month postoperatively (*P* < 0.0001, Fig. 2a). Notably, the operative eyes were not always the ones exhibiting extorsion. To ensure consistency with the analysis of the intorsion group and eliminate bias resulting from both eyes exhibiting extorsion, we specifically selected 8 patients with unilateral extorsion and the operative eyes demonstrating extorsion for subsequent analysis. However, our analysis did not uncover any correlation between preoperative DFA of eyes having extorsion and angle of deviation or duration of strabismus. Interestingly, Pearson correlation analysis suggested near exodeviation reduction was negatively correlated with DFA reduction at postoperative 1 month (r = − 0.793, *P* = 0.0189, Fig. 2b).

**Figure 3:**
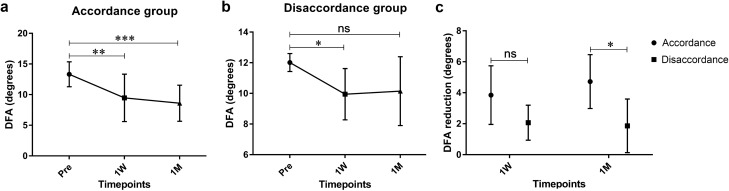
Ocular extorsion in accordance and disaccordance groups. (**a**) Longitudinal changes in DFA of the eyes exhibiting extorsion in accordance group. (**b**) Longitudinal changes in DFA of the eyes exhibiting extorsion in disaccordance group. (**c**) Comparisons in DFA reduction between accordance and disaccordance groups at 1 week and 1 month after surgery. *P* values for comparisons relative to preoperative level: RM one-way ANOVA with Dunnett’s multiple comparisons test. *P* values for comparisons between accordance and disaccordance groups: independent *t* test. (ns *P* > 0.05, *P < 0.05, **P < 0.01, ***P < 0.001).

To further assess the surgical effect on ocular extorsion, we opted to examine a sample of 14 patients with unilateral extorsion, as opposed to the 4 patients with bilateral extorsion, due to the potential divergence in their pathogenesis. These 14 patients were subsequently categorized into two groups, namely the accordance and disaccordance groups, based on the correspondence between the operative eye and the eye exhibiting extorsion. There were no significant differences observed between the accordance and disaccordance groups across preoperative parameters (all *P* > 0.05, Supplementary Table S1). These parameters included age at surgery, gender, duration of strabismus, spherical equivalent refraction, sensory fusion, stereoacuity, angle of deviation, and DFA. For the accordance group, the mean DFA of eyes having extorsion decreased from a preoperative value of 13.3 ± 2.0° to 9.5 ± 3.9° at 1 week (*P* = 0.0013) and 8.6 ± 2.9° at 1 month postoperatively (*P* = 0.0002, Fig. 3a). For the disaccordance group, the mean DFA reduced from the preoperative value of 12.0 ± 0.6° to 10.0 ± 1.7° at 1 week postoperatively (*P* = 0.0115), but remained relatively unchanged to 10.2 ± 2.2° at 1 month postoperatively (*P* = 0.0781, Fig. 3b). The DFA reduction of accordance group was comparable to that of disaccordance group at 1 week postoperatively (*P* = 0.0642, Fig. 3c). The DFA reduction of accordance group was larger than that of disaccordance group at 1 month postoperatively (*P* = 0.0101, Fig. 3c).

Among patients without ocular torsion, the preoperative degree of torsion in the operative eyes did not correlate with amount of exodeviation, stereoacuity or strabismus duration. Furthermore, the preoperative level of ocular torsion was comparable to the postoperative levels.

## Discussion

This study enrolled 72 patients with IXT who underwent R&R to evaluate ocular torsion. To our knowledge, this is the first study examining alterations in objective ocular torsion following R&R. Both exodeviation and ocular torsion was improved postoperatively. The improvement in extorsion was more pronounced when the operation was performed on the eye exhibiting extorsion.

In our study, the preoperative DFA of 72 patients with IXT was 6.8 ± 4.0°, which aligned with findings from previous research. Kang et al.^15^ reported DFA for the right and left eyes of 44 IXT patients aged 3–82 years as 5.70 ± 3.35° and 6.37 ± 3.36°, respectively, using fundus photography, and 5.73 ± 3.61° and 6.16 ± 3.50° using OCT. Shin et al.^8^ calculated a mean DFA of 6.13 ± 4.16° in 150 IXT children aged 4–15 years, which was significantly higher than that in their normal control group. Similarly, the mean DFA in our study was larger than that in our normal population set. Qiu et al.^16^ observed a mean DFA of 5.5 ± 3.1° in 138 eyes from 138 healthy myopic subjects aged 18–40 years, and Guo et al.^17^ examined 382 school children aged approximately 6.4 years and reported a mean DFA of 5.2 ± 4.0°.

In our study, OCT revealed ocular torsion in at least one eye of 25 IXT patients (34.7%). Similarly, Shin et al.^8^ reported ocular torsion in at least one eye in 30% of IXT patients. Additionally, Khanna et al.^18^ found that 59% of patients with infantile esotropia exhibited ocular torsion. These findings indicate that objective ocular torsion is not exclusive to cases of cyclovertical strabismus but also occurs in horizontal strabismus. The presence of ocular torsion may result in alphabet patterns and abnormal elevations or depressions in adduction^19^. Thus, it is crucial to consider and address any potential abnormal ocular torsion in cases of horizontal strabismus.

This study observed a significant reduction in the incidence of ocular torsion following surgery. Quantitative analysis further revealed that DFA significantly decreased postoperatively in eyes with extorsion compared to preoperative level, while in eyes exhibiting intorsion, the DFA increased significantly after surgery. These findings are consistent with previous research. For instance, a study involving 60 children aged 3–14 years with IXT accompanied by ocular torsion demonstrated a significant decrease in the degree of ocular torsion after lateral rectus recession^9^. Similarly, Khanna et al.^18^ investigated the effect of unilateral horizontal rectus surgery on objective ocular torsion in 68 children aged 4–16 years with congenital esotropia. They also reported a significant decrease in the incidence of ocular torsion after surgery, with a significant decrease in DFA in eyes with extorsion and a significant increase in DFA in eyes with intorsion postoperatively^18^.

The presence of ocular torsion in IXT patients and its relief after horizontal rectus surgery can be explained by several hypotheses. Kushner et al.^20^ proposed that ocular torsion in IXT may arise from changes in the oblique muscles. Shin et al.^8^ believed that prolonged ocular exodeviation could lead to relaxation and shortening of the oblique muscles, resulting in an imbalance of tension, thereby causing ocular torsion in the primary position. Consequently, after corrective surgery for exotropia, the tension imbalance of the oblique muscles may be alleviated, partially restoring ocular torsion in the primary position. Considering that our study excluded patients with oblique muscle paralysis and A/V patterns, we speculate that the tension imbalance described by Shin et al.^8^ may primarily affect the torsional movement of the eyeball, but has not yet reached the severity to cause muscle paralysis or vertical movement disorders. This is consistent with the findings of Bdeer et al.^21^, who observed that in the presence of inferior oblique overaction, ocular torsion was common in children with horizontal strabismus, even without V pattern. On the other hand, Deng et al.^22^ found that more than 20% of subjects had ocular torsion without accompanying oblique muscle dysfunction, suggesting that ocular torsion may not solely stem from oblique muscle abnormalities. As noted in other studies, defective binocular fusion and subsequent disruption of cyclofusion may also contribute to ocular torsion^8,23^. Effective correction of exotropia through R&R improves both binocular fusion and cyclofusion, naturally leading to a recovery of ocular torsion. As demonstrated in our study, the number of patients with ocular torsion decreased over time after surgery. This may be attributed to the gradual restoration of binocular fusion, leading to a gradual relief of ocular torsion. Additionally, MRI studies supported the hypothesis that ocular torsion was caused by abnormal pulleys or misalignment of extraocular muscles^24,25^.

Our study revealed a close relationship between IXT and intorsion. Specifically, there was a positive correlation between the severity of IXT and the degree of intorsion preoperatively. There was also a positive association between the correction of exodeviation and the improvement of intorsion, indicating that as exodeviation was corrected, intorsion was also improved. Thus, we speculate that exodeviation plays a crucial role in the pathogenesis of ocular intorsion. Since R&R is an effective approach for correcting exotropia, it naturally leads to a significant improvement in ocular intorsion. However, in patients with ocular extorsion, we did not observe a significant correlation between the severity of IXT and the degree of extorsion before surgery. More interestingly, we found a negative correlation between the correction of exodeviation and the improvement in extorsion. This suggested that exodeviation may primarily enhance the stability of extorsion rather than directly determine its severity. In other words, the greater the degree of exodeviation, the stronger the tolerance of extorsion to R&R seems to be. Therefore, for patients with large-angle exotropia, although exotropia is improved after R&R, this may not necessarily lead to a greater improvement in extorsion.

Our study revealed that the accordance group showed greater improvement in extorsion compared to the disaccordance group. This suggested that the efficacy of postoperative correction of extorsion was greater when the operation was directly performed on the affected eye, which was similar with the findings reported by Lee et al.^26^. The significant change in ocular extorsion observed in the accordance group postoperatively may be attributed to surgery-induced alterations in extraocular muscle tension. Conversely, the postoperative change in ocular extorsion observed in the disaccordance group was more likely a secondary adaptive response by the eye to changes in the rotational state of the operative eye. This secondary adaptive change may not be sufficient to significantly alter ocular extorsion^26^. Therefore, preoperative assessment of ocular torsion and performing R&R on the eye exhibiting torsion are essential for more effective correction of ocular torsion.

This study failed to establish a correlation between the severity of strabismus and DFA in patients without ocular torsion, potentially due to the limited sample size and confounding factors, such as age and refractive error. It is worth noting that DFA has been shown to have a positive association with age^27,28^. Additionally, there was an increase in extorsion observed in patients with uncorrected myopia and astigmatism^29^. However, some researchers have reported that DFA was not associated with age or refractive error in children^17,30^. Future studies should investigate the influence of age and refractive error on ocular torsion in IXT children. Furthermore, this study found no significant difference between preoperative and postoperative DFA of operative eyes among patients without torsion, suggesting R&R had limited impact on ocular torsion in this particular subgroup of patients.

There are some limitations to note in our study. First, only 72 patients were enrolled. Considering the small sample size, the subgroup analyses may be underpowered. Second, the patients were followed up for only one month, when they did not recover completely from the surgery^31,32^. Consequently, alterations in ocular torsion might occur beyond this period. The limited one-month follow-up period may not be adequate to capture all the changes. Therefore, it is imperative to explore the enduring effects of surgery on ocular torsion and the association between surgical outcomes and ocular torsion. Future studies with extended follow-up periods may yield crucial insights. Finally, the analysis of ocular torsion changes was limited to those occurring after R&R. Further studies are necessary to investigate the alterations in ocular torsion after other types of strabismus surgery, such as unilateral lateral rectus recession-medial rectus plication, and to compare their effects on ocular torsion.

In conclusion, ocular torsion in IXT patients could be observed and subsequently improved following R&R, particularly in case where the surgery was performed on the eye exhibiting torsion. It is recommended that preoperative assessments of objective torsion be conducted and that R&R be performed on the eye exhibiting torsion, if present, in order to more effectively correct ocular torsion in IXT patients.

## Patients and methods

This research was an observational, prospective, and single-center study involving patients with IXT. It was conducted in the Second Hospital of Dalian Medical University between March and June 2023. The study received approval from the Ethics Committee and was conducted in compliance with the principles of the Declaration of Helsinki. Prior to participation, written informed consent was obtained from the parents or legal guardians of the participants. The study’s inclusion criteria comprised of patients aged 5 to 18 years who were diagnosed with IXT and underwent R&R. The surgery was performed on the non-dominant eye. All patients were operated by the same strabismus surgeon (Qi Zhao). The exclusion criteria included individuals with myopia (≤ − 6.0 diopters) or astigmatism (≥ 3.0 diopters), any neurologic disease, nystagmus, amblyopia, oblique paralysis, alphabet pattern strabismus, any organic eye disease, orbital pathology, history of ocular trauma or surgery, and non-cooperation. The flow chart depicted the enrollment of participants in the study (Supplementary Fig. S2).

### Baseline and follow-up

Patient characteristics, including sex and age at surgery, were obtained from medical records. Baseline examinations were conducted prior to surgery. Subsequently, follow-up examinations were performed at 1 week and 1 month after surgery. These examinations comprised of prism and alternate cover test to evaluate angle of deviation, Worth 4-dot test to assess sensory fusion, Titmus test to determine stereoacuity, and OCT (Carl Zeiss Meditec, Inc.) to assess objective ocular torsion. Prism and alternate cover test was conducted at both 30 cm and 6 m distances while the participants were in primary gaze with appropriate spectacle correction. The angle of exodeviation was recorded as a positive value, while the angle of esodeviation was documented as a negative value.

### Evaluation of objective ocular torsion by OCT

**Figure 4:**
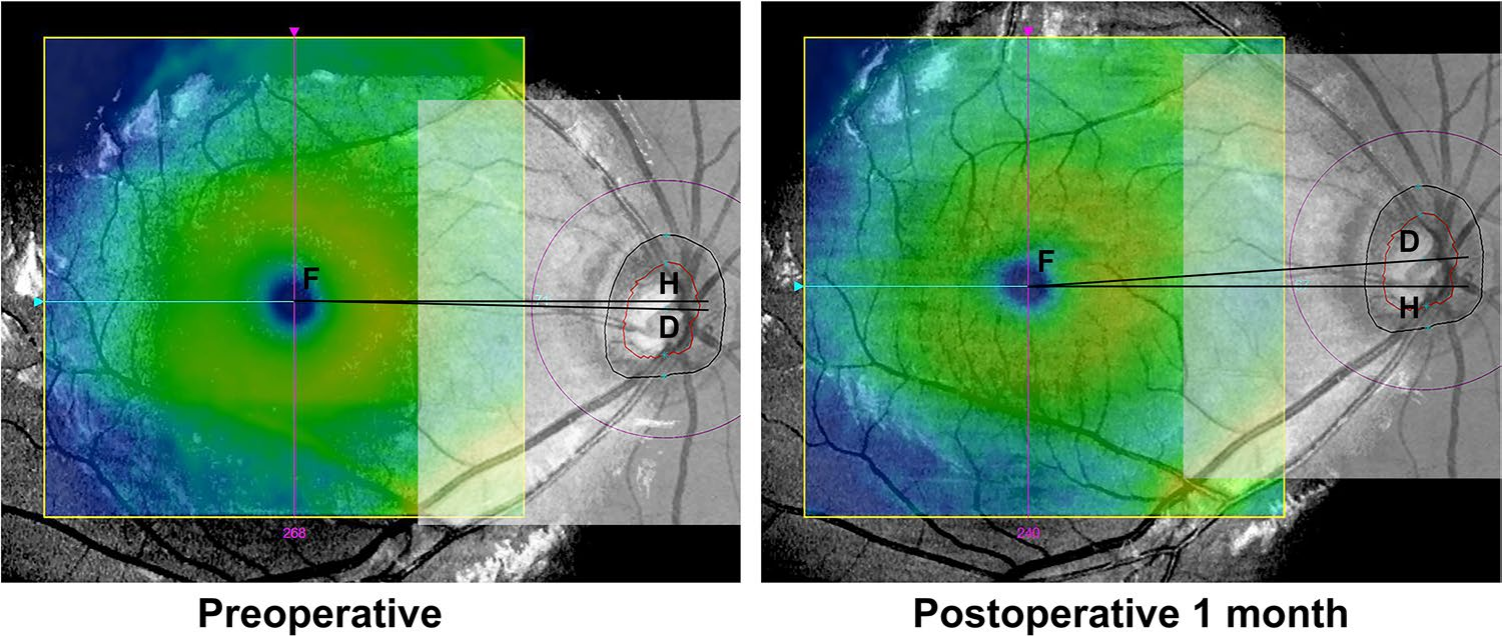
A representative picture of preoperative (left) and postoperative (right) DFA calculation. The fovea and optic disc center were automatically detected on scanning laser ophthalmoscopy fundus and retinal nerve fiber layer thickness deviation images, respectively. These images were then manually co-registered using Adobe Photoshop CC software. The DFA was calculated as the angle between the horizontal line and the disc-fovea line on the co-registered image. This eye improved from preoperative intorsion to having no torsion at 1 month postoperatively.

During the acquisition of OCT imaging, we carefully checked head position and avoided any head tilt to minimize the potential bias in measurement resulting from head position. Objective ocular torsion was assessed by measuring DFA as previously described^16^. Briefly, fovea was automatically identified on scanning laser ophthalmoscopy fundus image and optic disc center was automatically detected on retinal nerve fiber layer thickness deviation image using OCT software. Subsequently, the two images were manually co-registered with Adobe Photoshop CC software (Adobe Systems Inc.). The DFA was calculated as the angle between the horizontal line and the disc-fovea line on the co-registered image (Fig. 4). A positive DFA value indicated an inferior location of the fovea relative to the optic disc center, while a negative value indicated a superior location.

For qualitative assessment of objective torsion, eyes were classified into distinct categories based on established criteria^18,33,34^. Specifically, one eye was identified as no torsion if the fovea was situated within a region defined by the center and bottom edge of the optic disc, as displaying intorsion if the fovea was located above the center of the optic disc, and as demonstrating extorsion if the fovea was positioned below the bottom edge of the optic disc.

### Statistical analyses

Statistical analyses were conducted using GraphPad Prism 7.0 (GraphPad software Inc.) and SPSS 22 (SPSS Inc.). Continuous variables were presented as mean ± standard deviation, and categorical variables were presented as number (%). For comparisons between two data sets, independent *t* tests or nonparametric tests (Mann–Whitney *U*) were employed depending on the normality of the data as determined by normality tests. For comparisons involving three or more data sets, a repeated measures one-way ANOVA with Dunnett’s multiple comparisons test was used. Fisher’s exact tests were used to compare categorical variables. Pearson correlation analysis was used to calculate the correlation coefficient. Statistical significance was determined by a *P* value less than 0.05.

### Supplementary Information

Supplementary Information.

## Data Availability

The datasets generated during and/or analyzed during the current study are available from the corresponding author on reasonable request.
