# Supplementary material for: The changes in ocular torsion after unilateral lateral rectus recession-medial rectus resection for intermittent exotropia
Source: Sci Rep. 2024 Jun 21;14:14300. doi: 10.1038/s41598-024-65193-z (PMC11192914; doi:10.1038/s41598-024-65193-z)
Supplement: Supplementary file 1 — Supplementary Information. [file 41598_2024_65193_MOESM1_ESM.pdf]

The changes in ocular torsion after unilateral lateral rectus recession-medial rectus resection for intermittent exotropia

Changyang Liu[1], Jiasu Liu[1], Huailin Zhu[1], Lan Zhang[1], Mingjun Gao[1], Siqi Zhang[1], Qi Zhao[1*]

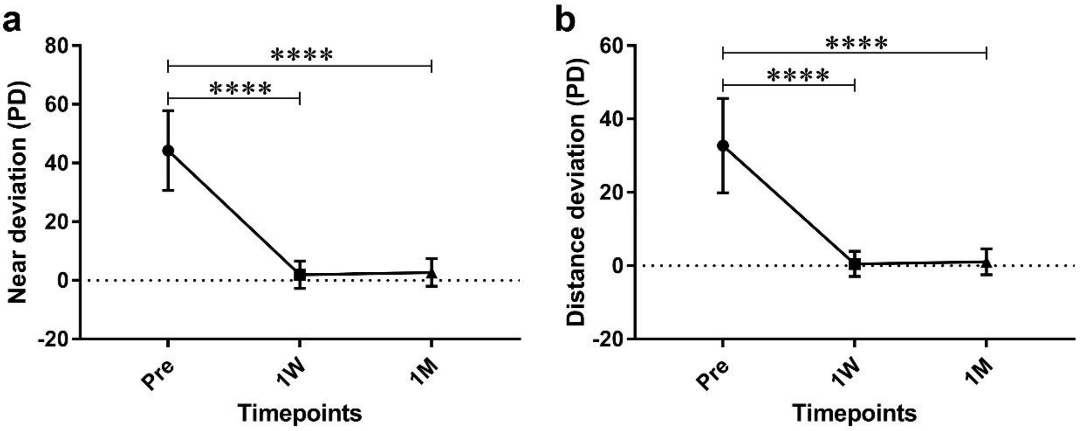

Supplementary Fig. S1 Corrected exodeviation after unilateral lateral rectus recession-medial rectus resection (R&R). **a** Longitudinal changes in near deviation. **b** Longitudinal changes in distance deviation. PD, prism diopters; **** P <0.0001; repeated measures (RM) one-way ANOVA with Dunnett's multiple comparisons test.

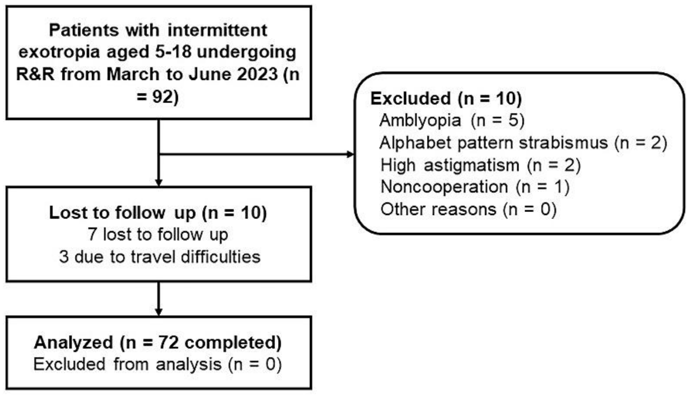

Supplementary Fig. S2 Flow chart of participants enrolled in this study.

Supplementary Table S1 Comparisons of demographic and clinical characteristics between accordance and disaccordance groups

Variable	Accordance group (n = 8)	Disaccordance group (n = 6)	*P* value
Age at surgery (years)	7.9 ± 2.0	10.0 ± 2.7	0.1164 [a]
Gender (male/female) (number)	5/3	2/4	0.5921 [b]
Strabismus duration (years)	1.9 ± 2.4	2.8 ± 2.7	0.5065 [c]
Mean SER of both eyes (diopters)	-0.92 ± 1.78	-1.33 ± 1.04	0.6251 [a]
Fusion (normal/abnormal) (number)	7/1	6/0	1.0000 [b]
Good preoperative stereoacuity (≤ 100 arcsec) (number)	4/4	3/3	1.0000 [b]
Preoperative amount of deviation at near (PD)	39.4 ± 6.1	46.6 ± 13.9	0.2119 [a]
Preoperative amount of deviation at distance (PD)	27.5 ± 6.4	34.7 ± 17.1	0.3664 [a]
Preoperative pathological extorsion (degrees)	13.3 ± 2.0	12.0 ± 0.6	0.1200 [a]

Abbreviations: SER, spherical equivalent refraction; PD, prism diopters.

[a] Independent *t* test

[b] Fisher's exact test

[c] Mann-Whitney U test
